# TOB-STOP-COP (TOBacco STOP in COPd trial): study protocol—a randomized open-label, superiority, multicenter, two-arm intervention study of the effect of “high-intensity” vs. “low-intensity” smoking cessation intervention in active smokers with chronic obstructive pulmonary disease

**DOI:** 10.1186/s13063-020-04653-z

**Published:** 2020-08-21

**Authors:** Mohamad Isam Saeed, Pradeesh Sivapalan, Josefin Eklöf, Charlotte Suppli Ulrik, Charlotta Pisinger, Therese Lapperre, Philip Tønnesen, Nils Hoyer, Julie Janner, Marie Lavesen Karlsson, Charlotte Sandau Bech, Kristoffer Marså, Nina Godtfredsen, Eva Brøndum, Birgit Munk, Merete Raaschou, Andrea Browatzski, Pernille Lütken, Jens-Ulrik Stæhr Jensen

**Affiliations:** 1 Department of Internal Medicine C, Section of Respiratory Medicine, Herlev and Gentofte University Hospital, Hellerup, Denmark; 2 Department of Internal Medicine, Zealand University Hospital, Roskilde, Denmark; 3 Department of Respiratory Medicine, Hvidovre University Hospital, Hvidovre, Denmark; 4 Center for Clinical Research and Prevention, Bispebjerg and Frederiksberg Hospital, Frederiksberg, Denmark; 5 Department of Respiratory Medicine, Bispebjerg and Frederiksberg Hospital, Copenhagen, Denmark; 6 Department of Respiratory Medicine, Rigshospitalet, Copenhagen, Denmark; 7 Department of Respiratory and Infectious Medicine, Nordsjællands Hospital, Hillerød, Denmark; 8 Palliation Unit, Herlev and Gentofte University Hospital, Herlev, Denmark; 9 Rygestopcaféen, Hvidovre University Hospital, Hvidovre, Denmark; 10 Patient Representative, Hellerup, Denmark; 11 Rygestopkonsulenterne ApS, Hillerød, Denmark; 12 PERSIMUNE & CHIP, Department of Infectious Medicine, Rigshospitalet, Copenhagen, Denmark

**Keywords:** COPD, Randomized controlled trial, Smoking cessation, Exacerbations, Varenicline

## Abstract

**Background:**

Cigarette smoking is the leading cause of chronic obstructive pulmonary disease (COPD), and it contributes to the development of many other serious diseases. Smoking cessation in COPD patients is known to improve survival and reduce the number of hospitalization-requiring acute exacerbations of COPD. However, smoking cessation interventions in these patients have only been successful for approximately 15–20% for consistent smoking abstinence in 12 months. Thus, more effective interventions are needed for this patient group. The aim of this study is to determine whether a high-intensity intervention compared to a low-intensity intervention can increase the proportion of persistent (> 12 months) anamnestic and biochemical smoking cessation in active smokers with COPD.

**Methods:**

This study is a randomized controlled trial. A total of 600 active smokers with COPD will be randomly assigned 1:1 to either a standard treatment (guideline-based municipal smoking cessation program, “low intensity” group) or an intervention (“high-intensity” group) group, which consists of group sessions, telephone consultations, behavior design, hotline, and “buddy-matching” (smoker matched with COPD patient who has ceased smoking). Both groups will receive pharmacological smoking cessation. The primary endpoint is anamnestic and biochemical (cotinine analysis in urine) validated smoking cessation after 12 months.

**Discussion:**

The potential benefit of this project is to improve smoking cessation rates and thereby reduce smoking-related exacerbations of COPD. In addition, the project can potentially benefit from increasing the quality of life and longevity of COPD patients and reducing the risk of other smoking-related diseases.

**Trial registration:**

ClinicalTrials.gov NCT04088942. Registered on 13 September 2019

## Background

COPD is a life-threatening and incurable lung disease characterized by persistent breathing problems and poor airflow in the lungs that usually worsens over time. Globally, it is estimated that 250 million people have COPD and that three million deaths annually, corresponding to 5% of all deaths, are caused by COPD [1]. In Denmark, COPD is the third most common cause of death, as it contributes to 5500 deaths a year [2]. Cigarette smoking is by far the most important cause of COPD by damaging the alveolar sacs (the tar and gas phases of cigarette smoking contain high concentrations of free radicals, leading to oxidative stress and inflammation [3]). Cigarette smokers have a higher incidence of respiratory symptoms and abnormal lung functions, a greater annual decline in FEV_1_ (forced expiratory volume in 1 s) and a greater mortality rate than non-smokers [4]. Other types of tobacco, e.g., pipes, cigar, hookahs, are also risk factors for the development of COPD [4, 5] as well as passive exposure to cigarette smoke (passive smoking) by increasing the overall burden on the lungs of inhaled particles and gasses [4].

Acute exacerbation of COPD is a worsening of the patient’s respiratory symptoms, such as shortness of breath and cough that lasts for several days, and requires medical treatment beyond the patient’s usual medicine. Exacerbations of COPD can be caused by tobacco smoking [6] and are associated with increased risk of mortality due to decreasing lung function and activity level and often lead to hospitalization [7, 8]. This is one of the biggest costs for the healthcare system for the treatment of COPD [9]. In addition, there is evidence that exacerbations of COPD increase the risk of myocardial infarction [10] and strokes [11]. Tobacco smoking in patients with COPD can also contribute to other comorbidities such as lung cancer (both with regard to individual and population risk [12]) that can contribute to the poorer overall health and increased risk of mortality. The individual risk of lung cancer increases with an increasing number of cigarettes smoked per day and the duration of years of smoking, and the population risk increases with the incidence of current smokers [12].

In this way, smoking cessation will be the most effective intervention to stop the development of COPD, lessen the aforementioned risks associated with tobacco smoking, and increase survival and reduce morbidity [13]. Overall, tobacco smoking thus increases mortality and serious morbidity as well as symptoms in COPD patients, and smoking cessation should be the top priority in treating COPD.

### Current evidence of smoking cessation

There are a number of approaches for treating tobacco dependence, including behavioral therapy and pharmacotherapy, but smoking cessation is only successful in a proportion of patients with COPD, especially on a long-term basis [14, 15].

A Cochrane review from 2016 (16 studies, involving 13,123 smokers with COPD) shows among other things, from two studies, sublingual nicotine tablets and varenicline increased smoking cessation rates compared to placebo over twice as much, respectively, RR 2.60 (95% CI 1.29–5.24) and RR 3.34 (95% CI 1.88–5.92) [16]. Pooled results from two other studies also showed a positive effect of bupropion compared to placebo (RR 2.03 (95% CI 1.26–3.28)) [16]. By combining these four studies, there was high-quality evidence for the effect of pharmacotherapy plus high-intensity behavioral therapy compared to placebo plus high-intensity behavior therapy (RR 2.53 (95% CI 1.83–3.50)) [16].

In a study from 2008, smoking cessation rates were examined in a group of patients with COPD who participated in a 1-year smoking cessation program (*N* = 247) and were compared with a group of COPD patients who received normal care (*N* = 231) with follow-up 1 year and 3 years after starting the program [17]. The smoking cessation program included a 2-week period of admission to hospitals, group sessions where NRT and exercise were recommended/advised in, and in addition telephone calls with specially trained staff who gave feedback and support for smoking cessation throughout the year [17]. In the smoking cessation program, 52% of patients ceased smoking after 1 year and 38% after 3 years, correspondingly in the normal care group 7% after 1 year and 10% after 3 years [17]. However, the study is not in a controlled design, but the results are still inspiring.

Although many different methods of smoking cessation and several years of preventive action have been tried, they have all had very little effect. And this despite the many known harmful effects of continued smoking. Thus, more effective interventions are needed for this patient group.

The hypothesis in the study described here is that a person-adapted, multi-component, intensive smoking cessation intervention results in a higher incidence of anamnestic and biochemical smoking cessation after 12 months in people diagnosed with COPD and (as the last 8 weeks before inclusion in the study) are current daily smokers than a standard smoking cessation intervention with municipal smoking cessation offers and offers of varenicline.

## Methods

### Design

This is a randomized open-label, superiority, multicenter, 2-arm intervention study, in which it is examined if a “high-intensity” intervention causes fewer people (diagnosed with COPD) to smoke after 12 months than in a “low-intensity” intervention in people diagnosed with COPD. The effect on survival for 12, 24, and 48 months; incidence of COPD exacerbations; number of admissions for all causes; and cardiovascular admissions will also be analyzed at the same time.

Additionally, a few sub-studies will be conducted:

- Sub-study 1: We will examine the prevalence of depression/anxiety within 36 months in the “high-intensity” and “low-intensity” groups by means of questionnaires, by hospital admissions, and by recording the prescription of drugs for depression and anxiety.
- Sub-study 2: In a subset of patients who quit smoking from the “high-intensity” group (*n* = 50) and patients who do not quit smoking from the “low-intensity” group (*n* = 50), we are going to examine the potential changes in the respiratory microbiome within 6 months of smoking cessation attempt.

### Recruitment and inclusion

Recruitment takes place through advertisements and announcements in local newspapers, daily newspapers, via the Danish Lung Association and its member magazine, where the participants then per mail or telephone can contact trial staff (see under the “Data collection” section) and then receive the written participant information per mail or other ways if the participant wants this. Later, an information meeting with trial staff can be agreed on one of the participating departments’ cadastre, in a booked room that is approved for patient examination. The participant will be informed of the right to an assessor in the first contact, and the right will also be clarified in the written participant information. For the meeting, oral participant information will be provided by trial staff, and the aim is to ensure that trial staff do not have work during the recruitment and if they have, the work telephone is handed over to other staff during the meeting. After the meeting, a 24-h reflection period will be given, and informed consent will be obtained upon the signed consent declaration, provided that the participant will participate in the project.

The inclusion criteria are as follows:

- Age ≥ 50 years
- Competent and capable
- Have diagnosed COPD [*spirometry verified and evaluated by pulmonary specialist*]
- Current daily smoker [*minimum 1 cigarette daily*]
- Have smoked minimum 20 pack-years (1 pack-year = 20 cigarettes daily in 1 year)
- Want to or try to stop smoking
- Do not mind taking varenicline or NRT during the trial
- Are willing to give blood and urine samples according to the protocol

The exclusion criteria are as follows:

- Previously included in the trial
- Hospitalized with COPD exacerbation within the last 24 months
- Are associated with hospital outpatient clinic for COPD disease treatment
- Have FEV_1_ < 50%.
- Pregnancy/breastfeeding
- Life expectancy less than 1 year
- Severe linguistic problems or inability to give informed consent
- Severe mental illness that is not controlled with medication
- Active alcohol or substance abuse
- Active cancer disease*

*The person can participate if he or she has had a cancer disease that is now referred to as curative/radically treated. Basal cell carcinoma of the skin does not count as an exclusion criterion.

### Interventions

A total of 600 participants will be included with start-up 1 January 2020 and is expected to be completed 3 years later, January 2023. Participants are randomized (random allocation) 1:1 to a “low-intensity group” and a “high-intensity group,” stratified for age (> 65 years vs. ≤ 65 years) and number of daily cigarettes (> 10/day vs. ≤ 10/day), and blocked randomization through REDCap with blocks of varying sizes (4–8), which will not be disclosed. Participants, their data, and laboratory specimens will be assigned a coded identification number, ID, to maintain participant confidentiality. Offers of varenicline are the standard municipal smoking cessation intervention and thus our choice of comparator. Participants will be randomized to one of the following:

1. “Low-intensity group”: encouraged to quit smoking via own GP and varenicline is prescribed for 12 weeks. Encouraged to only adhere to this “low-intensity” intervention.
2. “High-intensity group”:
  - Varenicline for 12 weeks
  - Group sessions—in all 30 sessions divided into 6 months
    i. Preparation phase*: 5 sessions
    ii. Days 1–14: 5 sessions
    iii. Days 15–30: 5 sessions
    iv. Days 31–60: 5 sessions
    v. Days 61–90: 5 sessions
    vi. Days 90–180: 5 sessions

*During the preparation phase, participants are allowed to smoke; smoking cessation starts at day 1.

Group sessions are controlled by the following:

I. Respiratory nurse [mapping of different smoking patterns and different reasons for smoking. When is smoking the greatest? When in the process, smoking starts to fall. Dangerous situations regarding smoking relapses. Working closely with psychologist—see below. Initial focus on nicotine-craving and coping methods]
II. Respiratory physician [lung function, lung age, anatomy, physiology, pathophysiology of lung cancer, and COPD]. Focus on why smoking cessation is good. Is there anything you want to experience in your life that smoking can prevent? Either by death or because illness would prevent it?
III. Psychologist [in close cooperation with the nurse. Focus on behavior before smoking and how this behavior is slowed down at an early stage. Coping by smoking. Cognitive smoking cessation strategies. Handle digito-oral habit].
IV. Physiotherapist [training on how to improve general physical performance status based on individual training programs].
V. Dietician (focus on keeping weight (both ways), nutrition). Suggestions for what to eat at episodes of smoking craving.
  (c) Relationships and retention via these:
    i. A hotline is established which the “high-intensity group” can call.
    ii. Weekly calls to all participants in the project for 26 weeks, reminding of adherence. If the participant has not had a relapse, there will be calls at week 34 and week 42. If the participant has had a relapse, calls continue until relapse-free for 10 weeks, then week 34 and week 42.
  (d) “Buddy-arrangement”: participants who have completed the program and have become smoke-free are matched with new ones in the program. A meeting frequency of approx. every 7–14 days. The first participants are matched with outpatients from the COPD clinic who have ceased smoking.

### Assessments

**Fig. 1:**
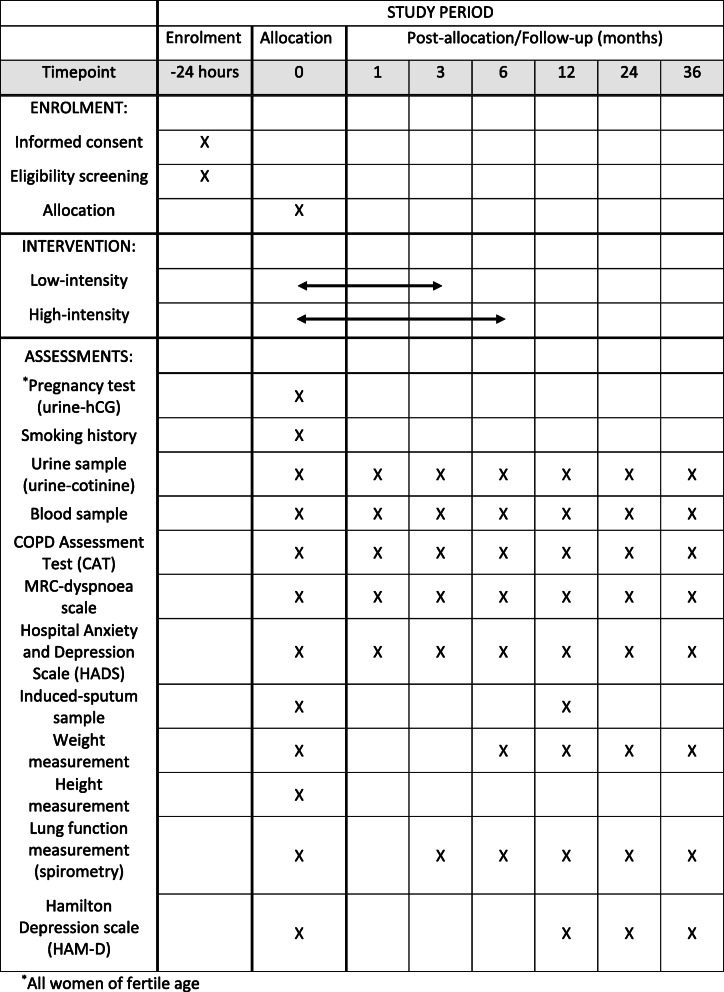
SPIRIT figure: overview of assessments that each participant will undergo and the time schedule for the project

The following assessments will be carried out during the project, cf. Fig. 1. Details of the low- and high-intensity interventions are described in the “Interventions” section.

#### Smoking history

The age of smoking debut is obtained, the number of years smoked, the number of cigarettes/cigars/cheroots/grams of tobacco smoked, and the number of smoking cessation attempts in total, if they have tried to stop smoking within the last year and motivation for smoking cessation.

#### Blood tests

The following are the standard blood samples: electrolyte parameters (sodium, potassium), kidney parameters (creatinine, carbamide), liver parameters (albumin, bilirubin, ALAT, alkalic phosphatase, INR), CRP, glucose, HbA1c, hematologic differential blood cell count, and hemoglobin and iron content.

#### Questionnaires

Standardized questionnaires are used. CAT and MRC dyspnea scale are short and simple tests that provide an understanding of the severity and impact of COPD on the participant’s daily life. HADS is used to screen for anxiety and depression, and HAM-D is used to suggest a risk of developing depression.

### Scientific ethics statement

The study is performed according to the Helsinki Declaration and is carried out in accordance with the rules in the Personal Data Act and the Health Act. Approval will be sought from the Danish Data Protection Agency. Recruitment and inclusion will take place as previously described. Participation requires a signed informed consent statement. Participants can withdraw their consent at any time and withdraw from the research project without affecting their right to current or future treatment. Participants who do not wish to participate in the trial will be offered treatment according to current guidelines. The participant also has the right to bring an advisor to the information conversation and is entitled to a reflection period before any consent form is signed. If, during the experiment, significant information emerges about the individual’s state of health, this will be disclosed both in written and orally to the participant, unless he or she has refused to do so in advance in the signed consent statement.

### Data collection

The primary daily project management is handled by the project manager. In addition, a project group (investigators), consisting of health professionals from the departments involved, is trained to assist the project manager with the recruitment, sampling, and follow-up of participants. Recruitment in general practice is done by the project-trained nurse. Assignments for project staff after patient allocation are detailed in standard operating procedures (SOPs), depending on the allocation to intervention or control group.

Upon entering the program and all visits in the future (after 1, 3, 6, 12, 24, and 36 months), the participant is summoned to a conversation with staff, asking if the participant smokes (yes vs. no), and if yes, how much. In addition, a urine sample is taken with cotinine analysis for biochemical verification of smoking status and standard blood samples to examine health status at these visits (analyzed on the Department of Clinical Biochemistry, KBA). In addition, questionnaires (CAT, MRC, and HADS) are completed with the participant.

In the case of inclusion and follow-up visits after 3, 6, 12, 24, and 36 months, lung function measurement is also carried out at regular intervals by spirometry. Smoking anamnesis and height measurement are done by inclusion in the study along with weight measurement, which is only further measured after 6, 12, 24, and 36 months to monitor BMI.

The HAM-D score is measured at inclusion, and again after 12, 24, and 36 months, and if the score becomes high (≥ 25), i.e., that there is a high risk of developing depression, the participant will be advised to see their GP for further evaluation.

In addition, sputum samples are induced for microbiome analysis at the time of inclusion into the study, and again after 12 months in the first 50 participants in the “high-intensity” group who quit smoking and in the first 50 participants in the “low-intensity” group who do not quit smoking.

Confirmation of death (including the cause of death) is obtained from the Cause of Death Register (DAR). Admissions, hospitalization, and the participant’s diagnoses are retrieved from their records. Comorbidities and prescribed therapy and filled prescriptions are recorded at inclusion and on an ongoing basis during study visits. During the trial, the following are also recorded: side effects and analyses from the paraclinical tests. At the same time, it is also stated that varenicline does not cause critical interactions with other drugs (see www.interaktionsdatabasen.dk and product summary).

Collected data will be treated confidentially by the staff associated with the project. Data will be reported in electronic case report forms (eCRF) specific to each participant, which are encrypted and stored in servers protected by Danish data security authorities with double login to REDCap (with automatic range checks during data entry to prevent unrealistic data entries). In addition to the above information, the following data are recorded by inclusion: demographic data, health status, current and previous drug consumption, current and past diseases. Physical copies of CRF are kept in locked archives for the departments involved for 15 years. A Good Clinical Practice (GCP) coordinator, independent from investigators and the sponsor, will continuously monitor the trial and ensure that recruitment, data entry, etc. have been done correctly.

### Information from patient journals

Information from the trial participants’ journals is obtained under the study to obtain study-relevant information (information to be used for the study-specific case report form or corresponding to the data to be obtained for subsequently approved additional protocols). If informed consent is obtained from the trial participants, it is permissible for the investigators to obtain this information from the patient record. Study-relevant information includes data on COPD severity, risk profile, disease development, and relevant co-morbidity to answer the hypothesis described. The informed consent also gives investigators, COP:TRIN, their representatives, and eventual monitoring authority direct access to obtain information in the participant’s journal, etc., including electronic journal, in order to see information about the participant’s health conditions, which are necessary in order to complete the research project and in monitoring purposes, including self-monitoring and quality monitoring, as these are required to be performed. No information from patient records is obtained until informed consent has been obtained from participants.

### Remuneration/services

No remuneration is paid to the study participants.

### Biological material

Blood samples are taken by venipuncture, and throughout the study period of up to 36 months, the blood drawn will be up to 120 ml of blood (a maximum of 18 ml per visit).

The collection of induced sputum will be done by mask inhalation with hypertonic saline solution [18]. Participants are thoroughly instructed by a respiratory nurse to expectorate into a container. Samples are frozen in − 80 °C freezer within 24 h.

### Research biobank

A research biobank is created for storing blood samples, urine samples, and sputum samples. In total, up to 120 ml of blood will be taken from each participant from inclusion to 36 months of follow-up. All samples are stored in a freezer in a locked room for analysis before the project ends. Microbiome analyses are performed on the sputum samples at Statens Serum Institut or another laboratory that has recognized expertise in microbiome analysis. All samples are kept in a pseudoanonymized form for 15 years, where they are analyzed and will be done in the years after recruitment is completed. This research biobank will be used to answer the hypotheses described in this protocol, including sub-studies. The research biobank ends at the end of the recruitment process, and all surplus material is transferred to a biobank for future research (see below). The analyses consist of standard blood sample analyses to check health status: electrolyte parameters (sodium, potassium), kidney parameters (creatinine, carbamide), liver parameters (albumin, bilirubin, ALAT, alkalic phosphatase, INR), CRP, glucose, HbA1c, hematologic differential blood cell count, and hemoglobin and iron content. Additionally, at inclusion, cotinine is analyzed in the blood for immediate analysis, and all excess material is transferred to the research biobank. The urine sample is analyzed for cotinine content for biochemical smoking status, and the sputum samples are examined for the microorganism composition (microbiome analysis).

#### Biobank for future research

Permission to create a new biobank for future research with the excess material will be sought from The Danish Data Protection Agency. Following the end of the project and the research biobank, the excess material will be transferred to this new biobank for future research within the COPD disease. The samples (the excess material from the research biobank) will be locked away and stored pseudoanonymized for 15 years in accordance with the current legislation including data protection laws. Informed consent to participate in the project permits biological material to be stored in the research biobank and the excess material in the biobank for future research.

### Risks, adverse effects, and events

When taking blood samples, transient discoloration of the puncture site is observed frequently (in 5–15%) due to a minor blood accumulation under the skin (bruising). In addition, there is a risk of slight pain during the insertion of the needle and minimal risk of infection.

There are no known risks and side effects associated with urine and sputum collection.

It is a general rule that all effective medications can also have side effects. For varenicline, mild nausea and abnormal dreams are the most common side effects. A complete list of all known adverse effects with varenicline is found at https://pro.medicin.dk/Medicin/Praeparater/4063.

It should be said that smoking cessation itself can give rise to several symptoms, and it may be difficult to distinguish between these symptoms and the side effects of the treatment. The summary of product characteristics of varenicline is used as a reference document when assessing whether a serious adverse reaction (SAR) is expected/unexpected and thus may be a suspected unexpected serious adverse reaction (SUSAR).

The investigator must report all serious incidents/side effects (SAR) to the sponsor as soon as possible. Thus, the sponsor can immediately report on to the Danish Medicines Agency and the Danish Committee on Health Research Ethics if it is deemed to be a SUSAR. In case of fatal or life-threatening side effects, this must be recorded and reported to the National Board of Health within 7 days of the sponsor being aware of such a suspected adverse reaction. Within 8 days of the report, the sponsor must notify the National Board of Health of all relevant information about the sponsor’s and investigator’s follow-up to the report. All other SUSARs must be reported to the Danish Medicines Agency no later than 15 days after the sponsor has gained knowledge of these. At the same time, test managers are informed at the other centers.

An annual list of all SUSARs that have occurred during the trial period is prepared and a report on the safety of the participants is submitted to the National Board of Health. In addition, all adverse reactions and events are reported at the end of the trial in the final report to the National Board of Health.

### Exclusion from or interruption of trial

If the physician responsible for the study considers it necessary, the person may at any time terminate the study if there is a medical justification (e.g., development of allergy to medication), a safety risk, or other circumstances. However, this must be done in agreement with the coordinating investigator. The participant may at any time, as mentioned in the above paragraph, withdraw their informed consent and withdraw from the project. It will not have any consequences for further treatment.

### Information about compensation schemes

The trial is covered by the patient compensation scheme if, contrary to expectation, damages occur during the trial and by inclusion.

### Outcomes

#### Primary endpoint

- Anamnestic and biochemical* validated smoking cessation after 12 months (dichotomous, measured as change in score from baseline)

*Cotinine is analyzed in a urine sample with cutoff value at 200 ng/mL, as validated point prevalence for the last 7 days

#### Secondary endpoints

- Number of admissions for exacerbations of COPD or death within 12 months (continuous)
- Number of admissions for all causes or death within 12 months (continuous)
- Number of cardiovascular events^1^ within 12 months (continuous)
- Changes in CAT score from baseline over 12 months (categorical)
- Changes in FEV_1_ from baseline over 12 months (continuous)
- Changes in BMI^2^ from baseline over 12 months (continuous)
- Clinically relevant changes in HADS score from baseline over 12 months (categorical, change in category 0–7, 8–10, and 11–21 points)
- Occurrence of DNA from the following: *M. catarrhalis*, *H. influenzae*, and *P. aeruginosa* over 12 months^3^ (dichotomous, measured as change in test result from baseline)
- Changes in the total lung microbiome over 12 months^3^ (continuous, measured as change in the total composition of lung microbiome from baseline)
- Occurrence of smoking-related cancer^4^ within 12 months (dichotomous)
- Number of admissions requiring NIV treatment or admissions to intensive care or death within 12 months (continuous)
- Changes in status from MRC dyspnea score from < 3 to ≥ 3, over 12 months (dichotomous)

^1^Defined as cardiovascular death, acute myocardial infarction, or unstable angina pectoris [19].

^2^BMI loss more than 1 unit.

^3^These endpoints are only examined on the first 50 who stop smoking from the “high-intensity” group against the first 50 who do not stop smoking from the “low-intensity” group.

^4^Lung cancer, urothelial cancer, pancreatic cancer, esophageal cancer, pharyngeal cancer, laryngeal cancer, tongue cancer, oral cancer, and tonsil cancer.

#### Long-term (follow-up) endpoints

- Occurrence of depression within 36 months (dichotomous)
  1. Admission to psychiatry with depression as the primary diagnosis (dichotomous)
  2. New start of antidepressant treatment after baseline (dichotomous)
  3. Clinically relevant changes in HADS score over 36 months (categorical)
  4. Changes in status from HAM-D score from baseline over 36 months (categorical, change in category 13–17, 18–24, and 25–52 points)
- Number of days during antidepressant treatment from baseline over 36 months (continuous)
- Changes in FEV_1_ from baseline over 36 months (continuous)

### Analyses

Data will be analyzed (blinded) using intention-to-treat (ITT) principles, including all the data available, regardless of whether the intervention was completed or not. The aims of the ITT analysis are also to provide unbiased comparisons among the two study groups and to avoid the effects of potential study dropouts and protocol deviations. Patients in the intervention group will be compared to patients in the control group.

For the primary outcome, the percentage of patients who have ceased smoking in each group will be calculated and the two groups will be compared using logistic regression. For the dichotomous outcomes and the nominal outcomes, both presented as counts and percentages, Fisher’s exact test and chi-squared test will be used, and the timed dichotomous outcomes will be visualized through Kaplan-Meier plots. In addition, the ordinal outcomes (also presented as counts and percentages) will be analyzed with the Mann-Whitney *U* test. Continuous outcomes will be expressed as means, standard deviations, and interquartile ranges and will be analyzed with *t* tests if normally distributed and Mann-Whitney *U* tests if non-normally distributed. Furthermore, adjusted analyses will be performed with a multivariable Cox proportional hazards model, adjusting for baseline variables and calculating hazard ratio. In addition, the combined outcomes “number of admissions for exacerbations of COPD or death …” and “number of admissions for all causes or death …” will be analyzed in a generalized linear model. Data will be processed and analyzed in SAS with graphs generated therein and in other graph programs such as SPSS.

A Data Safety Monitoring Board, independent from sponsor and competing interests, will monitor the safety of the trial by conducting interim analyzes based on primary and secondary endpoints when half of the study population has completed the study, and thus recommend continuation or termination of the trial.

### Sample size (statistical power calculation)

Type 1 error limit (*α*) of 5%. Power (1-*β*) of 90%.

The maximum smoking cessation rate for the “low-intensity group” is expected to be 10% in 12 months. With the above-described person-adapted, multi-component, intensive smoking cessation intervention in the “high-intensity group”, ≥ 20% of participants are expected to cease tobacco smoking for at least 12 months. Thus, a 10% absolute improvement in the likelihood of smoking cessation is expected, in this case, equivalent to the relative risk of smoking cessation on 2.0. This gives a sample size of 532 (266 + 266) persons. To account for possible withdrawal from the study and lost to follow-up, 600 participants will be recruited for the project.

### Publication of results

The trial has been registered at ClinicalTrials.gov (NCT04088942). The results from the project will be published in a peer-reviewed journal regardless of whether they are positive, negative, or inconsistent with authorship according to the Vancouver recommendations: (a) substantial contributions to the conception or design of the work or the acquisition, analysis, or interpretation of data for the work; (b) drafting the work or revising it critically for important intellectual content; (c) final approval of the version to be published; and (d) agreement to be accountable for all aspects of the work in ensuring that questions related to the accuracy or integrity of any part of the work are appropriately investigated and resolved. If publication in a scientific journal is not possible, the results of the trial will be published in a report format, which will be made available via the Internet.

## Discussion

Patients with COPD fear acute exacerbations as it increases dyspnea, cough, and sputum and is associated with significant morbidity and mortality. Smoking cessation is the best way to mitigate the exacerbation and progression of COPD in active smokers with COPD; however, different methods of smoking cessation have been tried with very little effect. The potential benefit of this multi-component smoking cessation project is to prevent smoking-related exacerbations of COPD and thereby reduce logistics and costs of hospitalization and treatment of COPD. In addition, the project can potentially benefit from increasing the quality of life and longevity of COPD patients and reducing the risk of developing lung cancer and other smoking-related diseases. Possibly, the project may also cause “healthy” smokers to stop smoking. Based on this, we believe that the experiment is scientifically sound and that the trial participants will not be exposed to irresponsible risks.

## Trial status

This protocol is version 8 from 18 June 2019. Important protocol amendments will directly be communicated to all investigators, sponsors, registries, and committees. Recruitment of participants is anticipated to begin 1 January 2020 and planned to end by 1 January 2023.

## Supplementary information

**Additional file 1.** SPIRIT 2013 Checklist: Recommended items to address in a clinical trial protocol and related documents.

## Data Availability

The data from the TOB-STOP-COP study will be available once the study is completed. Applications for data require a formal application and will be decided upon by the board of the TOB-STOP-COP study group.
